# Using the multiple mini interview as an assessment strategy within the first year of a health professions curriculum

**DOI:** 10.1186/s12909-018-1203-5

**Published:** 2018-05-03

**Authors:** Michael D. Wolcott, Jacqueline M. Zeeman, Wendy C. Cox, Jacqueline E. McLaughlin

**Affiliations:** UNC Eshelman School of Pharmacy, University of North Carolina, 329 Beard Hall, Chapel Hill, NC 27599 USA

**Keywords:** Multiple mini interview, Capstone, Competency based medical education, Nonacademic, Professional competence

## Abstract

**Background:**

The multiple mini-interview (MMI) is a common assessment strategy used in student selection. The MMI as an assessment strategy within a health professions curriculum, however, has not been previously studied. This study describes the integration of a 5-station MMI as part of an end-of-year capstone following the first year of a health professions curriculum. The goal of the capstone MMI was to assess professional competencies of students and to offer formative feedback to prepare students for their upcoming clinical practice experiences. The purpose of this study was to evaluate the psychometric properties of an MMI integrated into a health professions curriculum.

**Methods:**

Five capstone MMI stations were designed to each evaluate a single construct assessed by one rater. A principal component analysis (PCA) was used to evaluate the structure of the model and its ability to distinguish 5 separate constructs. A Multifaceted Rasch Measurement (MFRM) model assessed student performance and estimated the sources of measurement error attributed to 3 facets: student ability, rater stringency, and station difficulty. At the conclusion, students were surveyed about the capstone MMI experience.

**Results:**

The PCA confirmed the MMI reliably assessed 5 unique constructs and performance on each station was not strongly correlated with one another. The 3-facet MFRM analysis explained 58.79% of the total variance in student scores. Specifically, 29.98% of the variance reflected student ability, 20.25% reflected rater stringency, and 8.56% reflected station difficulty. Overall, the data demonstrated an acceptable fit to the MFRM model. The majority of students agreed the MMI allowed them to effectively demonstrate their communication (80.82%), critical thinking (78.77%), and collaboration skills (70.55%).

**Conclusions:**

The MMI can be a valuable assessment strategy of professional competence within a health professions curriculum. These findings suggest the MMI is well-received by students and can produce reliable results. Future research should explore the impact of using the MMI as a strategy to monitor longitudinal competency development and inform feedback approaches.

## Background

Competency-based medical education (CBME) represents a core principle in the health professions [1, 2]; it establishes a robust system to monitor learner progress with respect to an explicit set of outcomes essential for functional practice in healthcare [3, 4]. Assessing competency development can present a significant challenge as it often necessitates comprehensive and multifaceted assessment [5, 6]. The assessment of professional competence, frequently described as noncognitive or nonacademic constructs, may include questionnaires, surveys, objective structured clinical examinations (OSCE), and self-assessment scales. These strategies target professional competence by evaluating specific attributes such as communication, empathy, and integrity [7, 8]. Effective integration of professional competency assessment into health professions curricula can be varied, inconsistent, and misaligned if not properly designed or implemented.

The multiple mini interview (MMI) is a popular assessment strategy at the point of admissions to evaluate professional competence and thus far it has not been explored as an assessment method beyond student selection [9]. OSCEs often serve as the choice strategy to evaluate competence within health professions curricula; however, we argue the MMI could be a feasible and advantageous alternative based on distinct differences between the two assessment methods. The MMI is structurally similar to OSCEs, which is to be expected as the MMI was initially characterized as an “admissions OSCE” [10]. In an MMI, students rotate among several stations, similar to their participation in an OSCE circuit, and in each room, they are expected to engage with an interviewer or actor who evaluates them on select criteria [9, 10].

The key difference between these assessment modalities is in their measurement models. First, the MMI is designed to include questions or scenarios that target noncognitive or nonacademic constructs (i.e. social and behavioral professional competence) [11]. Conversely, the OSCE generally focuses on the measurement of clinical knowledge and procedural skill development. These design differences correspond to variations in the evaluation approach criteria. In an OSCE, participants are required to complete a specific task (e.g. complete a physical exam, establish a diagnosis) that is evaluated with a pre-determined checklist of critical events that must occur. In an MMI, there may be an overall objective based on the scenario, (e.g. talk about a difficult situation, communicate an idea to someone); however, the focus is on the participant’s process and therefore includes a more holistic assessment of their approach. There are generally no outlined procedures to be completed or outlined expectations for the interaction.

The intent of the MMI, therefore, is to focus on professional competence. Conversely, the primary intent of the OSCE is to assess clinical competence. Frequently, OSCEs are designed to include evaluations of professional competence (e.g. communication, building rapport). However, using the OSCE alone to assess both clinical *and* professional competence can be problematic. Attempting to measure too much during a brief interaction can diminish the reliability and validity of results due to the inherent limitations of raters evaluating multiple constructs [10, 12]. The use of an MMI as an assessment strategy to evaluate professional competence helps mitigate these limitations in evaluating learner performance.

Limiting the MMI to selection contexts restricts and underutilizes this assessment strategy. At the point of admissions, for example, MMI participants are rarely (if ever) offered feedback about their performance. From the perspective of CBME, integrating MMIs into health professions curricula could serve as a mechanism for assessing and providing information to students about their current abilities and their professional growth starting from the point of candidacy. OSCE progress testing has been shown to provide valid and reliable data while supporting the assessment for learning paradigm through frequent evaluation of clinical competence [13–15]; therefore, it is plausible MMIs can offer a similar advantage when focusing on professional competency development.

Our goal was to determine if the MMI could be tailored to fit within a health professions curriculum. The purpose of this paper, therefore, is to describe the design and implementation of an MMI as part of an end-of-year capstone (C-MMI) with a focus on the integrity of the assessment results. We offer insights into the psychometric properties of the C-MMI, acceptability of the model, and discuss the utility of integrating MMIs into health professions curricula.

## Methods

### Capstone design

The C-MMI was one component of an end-of-year first-year capstone implemented at the University of North Carolina at Chapel Hill (UNC) Eshelman School of Pharmacy in spring 2016 as part of the transformed doctor of pharmacy (PharmD) curriculum [16]. The capstone was immediately preceded by the first full year of coursework and followed by the students’ first 8-week immersion experience into clinical practice (i.e. clinical rotations or clerkships). The first-year capstone was built to align with the School’s PharmD program core competencies and contained three parts: (1) a closed-book exam measuring retention of knowledge from first-year coursework, (2) an open-book exam aimed at determining the ability to synthesize and extend knowledge across multiple courses, and (3) the C-MMI measuring the proficiency of key professional competencies. The structure and evaluation for the C-MMI aligned with the School’s admissions MMI [17]. The results of the closed-book exam and open-book exam are published elsewhere [18].

The C-MMI was implemented over two half-days. On day one, students completed the teamwork MMI and were evaluated on two constructs: *giving instructions* and *receiving instructions*. At this station, students were provided two minutes to read a prompt before entering a room and rotating the role of *giving instructions* and *receiving instructions*. There were two evaluators in the room, one responsible for evaluating each student when giving instructions and one responsible for evaluating each student when receiving instructions. On day two, students completed three additional MMI stations evaluating *adaptability*, *integrity*, and *empathy*. Each station was designed to assess specific constructs of interests (i.e. targeted assessment) valued by the institution as outlined in the PharmD program outcomes and consistent with constructs assessed during the admissions MMI. Students were provided two minutes to read a prompt before entering the room where students then had six minutes to discuss the scenario with the interviewer. Each station had one interviewer who was responsible for evaluating the student according to the station’s construct of interest based on the student response to the scenario and student answers to a set of probing questions.

An essential purpose of the C-MMI was to provide formative feedback for students as they prepared for their early immersion experiences at the end of year one of the curriculum. Once the C-MMI was complete, students were provided a report categorizing their performance on the various constructs. A large group debriefing session was used to review overall performance on all three sections of the capstone as well as to offer strategies for continued knowledge and professional skill development.

### Data collection

At each station, students were evaluated on four criteria: (1) the construct of interest, (2) communication about that construct, (3) critical thinking, and (4) appreciation of the construct in pharmacy practice (i.e. understanding of the pharmacist’s role or context). Each criterion was measured on a scale of 1 to 10 ranging from “needs improvement” to “outstanding”. It was anticipated a 10-point scale would provide sufficient opportunities for raters to appropriately discriminate among students. A total maximum score of 40 was possible for each station. After the capstone, a survey was administered to assess student perceptions of the C-MMI assessment.

### Data analysis

Data were examined descriptively and are presented using mean ± standard deviation (SD). A principal component analysis (PCA) was conducted on the 20-item MMI with an orthogonal (varimax) rotation to evaluate the structure of the data and determine if the five constructs of interest were effectively measured [19]. We used Kaiser’s rule, which designated eigenvalues greater than 1 as the cut point for factor structure. Cronbachs’ alphas were calculated to evaluate the reliability of student performance data relating to the five constructs of interest, in addition to the intercorrelations of student performance at each station.

A three-facet Multifaceted Rasch Model (MFRM) was used to investigate student ability, rater severity, and MMI station difficulty. Scoring from the construct of interest was used to represent student performance at each station as it was confirmed to be an appropriate measure based on the factor analysis. FACETS Version 3.71.4 (Beaverton, Oregon) was used to analyze the three-facets simultaneously and independently to allow calibration onto a single, logit scale. Joint Maximum-Likelihood Estimation (JMLE) methods generated measures of student ability, rater severity, and station difficulty. The study included 148 students, 35 raters, and 5 stations, which produced a total of 740 ratings. The initial analysis included all data points obtained from the MMI with no missing data.

The results from the MFRM analysis provide Infit and Outfit Mean-Square (MnSq) error statistics. Large fit statistics are indicative of unexpected results, whereas small fit statistics suggest a lack of variability in observed ratings. Model fit control limits for the study were set at 0.5 for the lower limit and 1.7 for the upper limit [20]. Although fit statistics less than 0.5 are not ideal, they are not believed to distort the measurement system [20]. The results also include a mean-squares standardized statistic (Zstd) that reflects the randomness in the data. Absolute values greater than or equal to 2.0 suggest the rating is sufficiently improbable and requires further investigation for appropriate fit [20].

The goal of MFRM analysis was to derive a model that best accounted for student ability ratings based on the severity of the rater and the difficulty of the station. To optimize the fit of data to the model, students with Outfit MnSq statistics greater than or equal to 1.7 were closely examined and scores that appeared to be misaligned within the stations were removed (*n* = 37 ratings). A new analysis was conducted and the model was re-evaluated in an iterative process. The final MFRM analysis included a total of 703 ratings in which fifteen (2.1%) had Outfit MnSq statistics ranging from 1.7 to 2.1 but did not contain recognizable outliers. All student measurements had a Zstd statistics less than 1.5. The use of the fit statistic criteria aided in developing a best-fit model for evaluating student ability in the C-MMI.

To provide formative feedback to students, the capstone leadership decided translating raw scores into a performance category was the optimal method for reporting. For each construct, cut scores were selected based on the rubric’s pre-defined data points: *needs improvement* was assigned for scores less than or equal to 4; *satisfactory* for scores of 5 to 8; and *outstanding* for scores greater than or equal to 9. Student reports included the performance category for each construct and any feedback provided on the score sheet by the rater. This study was submitted and considered exempt from review by the Institutional Review Board of the University of North Carolina at Chapel Hill.

## Results

All first-year students (*n* = 148) completed 5 C-MMI stations. Sixty-eight percent were female, 62% were White, and 81% held a bachelor’s degree or higher. Raters (*n* = 35) were representative of the five academic divisions in the School. All raters previously served as raters for the School’s admissions MMI and were required to attend training the morning of the C-MMI assessments to ensure complete understanding of the goals, objectives, and assessment scales for their respective station. On average, students performed highest on the integrity and empathy stations (7.86 ± 1.66 and 7.22 ± 2.00 respectively) and lowest on the giving instructions and receiving instructions stations (6.20 ± 2.00 and 6.50 ± 1.73 respectively) (Table 1).

**Table 1:** Descriptive statistics of station performance and student (*n* = 148) classifications

	Teamwork^a^	Adaptability	Integrity	Empathy
Construct Score, mean (SD)	6.20 (2.00)	6.91 (2.08)	7.86 (1.66)	7.22 (2.00)
Needs Improvement, *n* (%)	22 (14.9)	27 (18.2)	6 (4.1)	17 (11.5)
Satisfactory, *n* (%)	109 (73.6)	81 (54.7)	88 (59.5)	90 (60.8)
Outstanding, *n* (%)	17 (11.5)	40 (27.0)	54 (36.5)	41 (27.7)

^a^Giving Instruction and Receiving Instruction construct scores averaged

### Factor analysis

The Kaiser-Meyer-Olkin (KMO) measure verified the sampling adequacy for the analysis with a KMO = 0.80. All KMO values for individual items were > 0.71, which is above the acceptable limit of 0.5 [19]. Bartlett’s test of sphericity indicated correlations between items were sufficiently large for PCA (***Χ***^2^ (190) = 1884.5, *p* <  0.001). Five factors exceeded Kaiser’s criterion of 1 and explained 76% of the variance. The factor model was determined to be a good fit based on diagonal values (0.98) and the proportion of residuals greater than 0.05 was 21.6%, well below the desired 50% [19]. The clustered items were consistent with the arrangement of variables in the MMI structure into 5 specific stations: giving instructions, receiving instructions, adaptability, integrity, and empathy (Table 2). The variables associated with each of the constructs were considered to be reliable with Cronbach alpha values all greater than 0.85 (range: 0.86–0.90) (Table 3). Giving instructions and receiving instructions were the most highly correlated (*r* = 0.45); all other correlations were equal to or less than 0.35, which further supports the constructs are separately targeted at each station (Table 3).

**Table 2:** Factor analysis loadings (principal component analysis with varimax rotation)

Factor	1	2	3	4	5
Station	Giving Instructions	Receiving Instructions	Adaptability	Integrity	Empathy
Construct	0.88	0.83	0.88	0.83	0.86
Communication	0.86	0.86	0.81	0.85	0.80
Critical Thinking	0.83	0.87	0.89	0.83	0.92
Pharmacy Appreciation	0.54	0.72	0.81	0.83	0.86
Eigenvalue	2.74	3.17	3.15	3.00	3.21
*% Variance Accounted For*	14	16	16	15	16

**Table 3:** Intercorrelations and reliabilities (Cronbach alpha) of C-MMI constructs

Station/Construct	1	2	3	4	5
1/Giving Instructions	(0.86)	0.45	0.14	0.02	0.13
2/Receiving Instructions		(0.88)	0.13	0.10	0.13
3/Adaptability			(0.90)	0.35	0.22
4/Integrity				(0.88)	0.14
5/Empathy					(0.90)

### MFRM

**Fig. 1:**
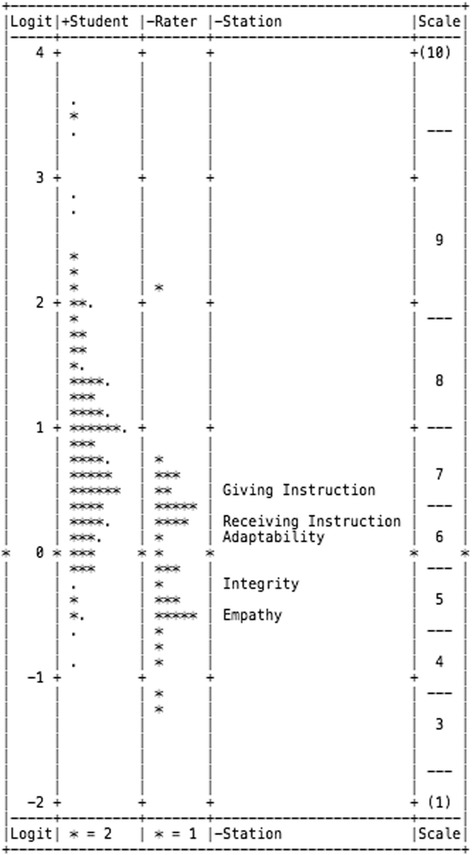
Variable map of student ability, rater severity, station difficulty, and scale performance. The highest performing students, most severe raters, and most difficult stations are located at the top of the diagram. All facets are positioned on a common interval log-odds scale

The MFRM explained 58.79% of the total variance in the ratings (Table 4), with most of this variance being attributed to differences in student ability (29.98%). Fifteen students had Outfit MnSq values between 1.70 and 2.08, but none of their ratings appeared to be anomalous outliers. As seen in Fig. 1, the variance of student ability ranged from 3.66 logits (highest performing student) to − 0.90 logits (lowest performing student). Most importantly, the reliability index of 0.77 suggests the students were reliably separated in their performance.

**Table 4:** Facet characteristics determined by MFRM analysis

Parameter	Student Ability	Rater Severity	Station Difficulty
Facet Explained Variance	29.98%	20.25%	8.56%
Mean Outfit MnSq	0.99	0.99	0.98
Model Sample RMSE (Standard Error)	0.40	0.21	0.07
Adjusted Standard Deviation	0.73	0.60	0.39
Separation	1.83	2.87	5.62
Reliability	0.77	0.89	0.97
Fixed Chi Square (*p*-value)	508.5 (< 0.01)	281.5 (< 0.01)	132.4 (< 0.01)

Differences in rater severity accounted for 20.25% of the variance in the data (Table 4). One rater (2.9%) had an Outfit MnSq score of 1.7 in addition to a Zstd score of 2.2, which suggests the rater assigned one or more ratings to students that were unexpected. In terms of severity, the rater was just below the average severity of all raters, which was considered acceptable for the purposes of this study. Only one rater (2.9%) had an Infit and Outfit MnSq score less than 0.5 with a Zstd of − 2.7, suggesting the rater used little variation in their pattern of ratings (Fig. 1).

Differences in station difficulty accounted for 8.56% of the variance in the data (Table 4). Stations had minimal variance with a range in difficulty from + 0.44 logits (most difficult) to − 0.55 logits (least difficult). The order of station difficulty from easiest to most difficult was empathy (− 0.55 logits), integrity (− 0.24 logits), adaptability (0.09 logits), receiving instruction (0.25 logits), and giving instruction (0.44 logits) (Fig. 1). The reliability index of 0.97 suggests the stations were reliability separated and chi-square indicates the stations were meaningfully separated with a high degree of confidence (*p* <  0.01) (Table 4).

Figure 1 provides a visual representation of the rating scale performance in which the horizontal dotted lines of the “Scale” column illustrate the category thresholds. These lines represent the point in which the likelihood of receiving the higher and lower category is equal. The goal is to have each category clearly separated from one another, as seen in Fig. 1. The overall performance of the rating scale is portrayed by the increasing average measure values when moving from the lower end of the rating scale to the higher end. The average measures increased as expected from − 0.99 to 2.14, which suggests students with higher ratings were displaying the construct more convincingly than those with lower ratings (Table 5) [20]. Outfit MnSq statistics for the rating scale are all located between the upper (1.7) and lower (0.5) fit limits, which further supports each of the categories functioned as intended.

**Table 5:** Rating scale characteristics

Category Labels	Number Times (%) Used	Average Measure	Outfit MnSq	Rasch-Andrich Threshold
1	2 (0)	- 0.99	0.9	
2	9 (1)	- 0.78	0.8	−2.35
3	22 (3)	- 0.60	0.7	−1.50
4	53 (8)	- 0.27	0.8	−1.24
5	68 (10)	0.06	0.8	−0.34
6	95 (14)	0.42	1.2	−0.12
7	135 (19)	0.81	1.1	0.20
8	159 (23)	1.07	1.0	0.77
9	114 (16)	1.73	1.0	1.72
10	46 (7)	2.14	1.1	2.86

### Student perceptions

Ninety-eight percent of students (*n* = 146) completed the post-capstone survey, which evaluated their perceptions regarding the C-MMI. Most students strongly agreed or agreed they took the Monday (i.e. teamwork) MMI (95.89%) and Tuesday (i.e. integrity, adaptability, empathy) MMI (97.26%) seriously and gave it their best effort. Most students also strongly agreed or agreed that the C-MMI allowed them to effectively demonstrate their communication skills (80.82%), critical thinking skills (78.77%), collaboration skills (70.55%), and knowledge of pharmacy (63.01%). Sixty-three percent of students strongly agreed or agreed they understood how to use feedback from the MMI in their upcoming clinical experiences (Table 6).

**Table 6:** Student (*N* = 146) perceptions of the capstone MMI

Survey Item	Strongly Agree N (%)	Agree *N* (%)
The MMIs allowed me to demonstrate my communication skills effectively	34 (23.29)	84 (57.53)
The MMIs allowed me to demonstrate my critical thinking skills effectively	34 (23.29)	81 (55.48)
The MMIs allowed me to demonstrate my collaboration skills effectively	29 (19.86)	74 (50.68)
The MMIs allowed me to demonstrate my knowledge of pharmacy effectively	13 (8.90)	79 (54.11)
I understand how to use feedback on my MMI performance during immersion	26 (17.81)	66 (45.21)

All items measured from 1 (strongly disagree) to 4 (strongly agree)

## Discussion

The MMI, to date, has been characterized exclusively as a methodology for summative purposes to rank candidates and inform selection decisions [21, 22]. Considering research generally suggests it offers fair, reliable, and valid data, the MMI presents a unique opportunity to evaluate students within the curriculum and collect data that could guide student professional competency development. The purpose of our study was to describe the design and implementation of an end-of-year capstone MMI specifically focusing on the quality of the assessment results. We designed the C-MMI using a targeted assessment approach in which prompts are tailored to address a specific construct of interest at each station, similar to MMI examples in the literature [11]. Our analyses support the use of the MMI as a reliable assessment strategy that can effectively be incorporated within health professions curricula to target the evaluation of select professional competencies.

The findings of this study are congruent with research demonstrating the validity and reliability of the MMI when used in selection contexts [11, 12, 23–25]. Our MFRM accounted for approximately 59% of the total variance in MMI scores, which is similar to previous studies using MFRM that account for 30–62% of total variance in performance data [11, 12, 23–25]. Similarly, variance attributed to candidate ability (30%) was within the range reported by other studies (16% to 45%) [11, 12, 23–25]. The C-MMI, therefore, appears to be a reasonable strategy to evaluate students reliably and provides valuable data in settings outside of admission.

Of note, variance associated with rater stringency was higher than anticipated at approximately 20%. This suggests the C-MMI raters introduced sources of construct-irrelevant variance due to leniency or severity in their ratings. Although raters were trained prior their participation and had previous experiences with the admissions MMI, the distribution of variance components suggests training should be reevaluated to ensure consistency among raters to minimize construct-irrelevant variance in subsequent studies. Specifically, one rater was identified as scoring students much lower compared to other raters (approximately 2 logits from the mean). Using MFRM, we are able to identify raters that may require targeted training that can minimize significant variation among raters. Additional training could include providing exemplars of student performance and their respective scores to help calibrate raters. Including descriptors for scale points may also help to reduce construct-irrelevant variance in this facet. Overall, this signifies the importance of appropriate training in the implementation of an MMI and its criticality regardless of the context.

The amount of variance associated with station difficulty was higher than other MFRM MMI studies, which report station difficulty representing 2–5% of the total variance [12, 23, 25]. The inclusion of the MMI into the curriculum presents a unique challenge to formulate questions and tasks that are relevant and more advanced compared to those used in admission contexts. This is a crucial element we identified which must be considered when integrating an MMI-type assessment within the curriculum. In admissions the context may be easily extracted, whereas when questions are posed within the curriculum, the topic becomes more contextualized. It is possible the difficulty among stations was not well-balanced as a result and questions in future iterations should be thoroughly reviewed for their relevance to the level of the learner. If there is a desire to minimize variance attributable to fluctuations in station difficulty compared to student ability, this could be a potential approach. In general, it demonstrates the significance of effective assessment design and supports the continuous piloting and improvements in the future.

A notable question when using the MMI is whether students would take the assessment seriously enough to produce data that adequately represent student ability. In an admissions environment, high-stakes are associated with their performance whereas the use within the curriculum may not instigate as much concern or motivation to fully participate when used as a formative assessment strategy. Based on the results of the survey, students agreed that they gave their best effort on the MMI stations and that the assessment allowed them to demonstrate various skills. Although the majority of students reported knowing how to use the feedback on their MMI performance during immersion, future revisions will ensure discussions with students about their performance is more detailed and provides specific instructions for improvement.

The data presented in this study reflect the first attempt to integrate the MMI into a health profession curriculum as an assessment strategy but also has several limitations. Notably, the first implementation of an assessment strategy limits the ability to evaluate its predictive validity, which can be an important focus for health professions curricula. For example, the application in admissions is intended to be predictive of later clinical and professional performance. In this study, we do not have data to suggest the predictive potential of the assessment. Furthermore, the use of MMIs later in the health professions curriculum may serve as additional predictors of performance after graduation, such as job performance evaluations, licensure and credentialing examinations, among others.

Next steps in this field of research should focus on evaluating how the C-MMI methodology could be used to monitor student growth over time and the impact of using MMIs as a formative assessment strategy. Although our purpose was to integrate the MMI as an assessment strategy intended to help the development of learners, we did not evaluate its function as a formative assessment tool. Instead, our focus was to describe the quality of the data that was obtained from the MMI when it is placed within a different context. With sufficient evidence, a C-MMI may also be considered an effective summative evaluation at the end of training to ensure learners are proficient in professional skill sets necessary for effective practice. Overall, the use of the MMI can contribute to competency assessment by providing valuable information for schools and learners regarding their professional skill sets.

## Conclusion

The MMI can be a valuable strategy in the comprehensive assessment of professional competencies when integrated within a health professions curriculum. We believe the findings presented here suggest the MMI can produce reliable results that can contribute to our understanding of a learner’s professional skill development. As this is the first example of the MMI being used as an assessment strategy within a curriculum, we hope it guides future studies that explore the approach as a method for formative, summative, longitudinal, and comprehensive assessment of professional competencies in health professions education.

## Abbreviations

CBME: Competency-Based Medical Education
C-MMI: Capstone Multiple Mini Interview
JMLE: Joint Maximum Likelihood Estimate
KMO: Kaiser-Meyer-Olkin
MFRM: Multifaceted Rasch Measurement (Many Facet Rasch Model)
MMI: Multiple Mini Interview
MnSq: Mean-Square
OSCE: Objective Structured Clinical Examination
PCA: Principal Component Analysis
SD: Standard Deviation
Zstd: Standardized Statistic
